# A comparative study of an NGO-sponsored CHW programme versus a ministry of health sponsored CHW programme in rural Kenya: a process evaluation

**DOI:** 10.1186/1478-4491-12-64

**Published:** 2014-11-05

**Authors:** Jackline O Aridi, Sarah A Chapman, Margaret A Wagah, Joel Negin

**Affiliations:** The Ford Family Program in Human Development Studies and Solidarity, University of Notre Dame, Nairobi, Kenya; Institute of Monitoring and Evaluation, Department of Organizational Psychology, University of Cape Town, Cape Town, South Africa; Development Concern International, Nairobi, Kenya; Sydney School of Public Health, University of Sydney, Sydney, Australia

## Abstract

The varied performance of Community Health Worker (CHW) programmes in different contexts has highlighted the need for implementation of research that focuses on programme delivery issues. This paper presents the results of process evaluations conducted on two different models of CHW programme delivery in adjacent rural communities in in Gem District of Western Kenya. One model was implemented by the Millennium Villages Project (MVP), and the other model was implemented in partnership with the Ministry of Health (MoH) as part of Kenya’s National CHW programme.

## Introduction

Community Health Worker (CHW) programmes have been utilized globally as part of primary health care approaches for many decades [1]. In most developing countries these approaches were strengthened in the late 1970s after the Alma Ata conference in 1978 that aimed at increasing access to health care through the call for ‘health for all’ [2]. Several decades on, the performance of CHW programmes has met with mixed reviews. Cochrane reviews have provided evidence that establish the effectiveness of CHWs in certain key areas such as exclusive breastfeeding, increasing immunization uptake and fewer children suffering from fever, diarrhoea and pneumonia [3]. However, other evaluations establish that in certain settings CHWs have been unable to decrease mortality and have provided poor quality services that were not consistent enough to substantiate impact [1, 4]. Clearly, while the use of CHWs has the potential to positively influence health outcomes of community members, there remain significant challenges in implementation particularly in national programmes when rolled out at scale [5].

With respect to challenges in implementation, a number of common barriers have been identified which include community-level factors such as health beliefs, geography and infrastructure as well as broader health system factors such as remuneration and supervision [6]. Irregular drug supply and inappropriate CHW recruitment have also been identified as factors that hinder implementation [7]. In other cases, there may be a strong local demand for curative services and not the health promotion services that CHWs routinely provide [8]. National-level CHW studies have identified four general problems: unrealistic expectations, poor initial planning, problems of sustainability and difficulty in maintaining quality [9, 10]. Yet, despite these challenges, it is still widely acknowledged that some role for CHWs is needed particularly in poor and underserved rural populations [11–13].

Given such varied performance of CHW programmes in different local contexts, calls for a comprehensive body of implementation research that focuses on CHW programme delivery issues are hardly surprising [14]. Implementation research, which includes process evaluations, can assist in the identification of barriers and facilitators that influence the adoption and uptake of ‘proven’ health technologies and interventions [15]. Within this type of analysis, we feel that the voices of CHWs themselves have the potential to greatly enrich our understanding of how implementation barriers are expressed and resolved in different contexts. Unfortunately, these voices are often conspicuously absent in CHW evaluation research literature [16].

Towards this aim, this paper presents the results of a process evaluation of a CHW programme implemented within the Gem (formerly Siaya) District in Western Kenya. The CHW model was formulated and delivered by the Millennium Villages Project (MVP), a development programme with operations across sub-Saharan Africa since 2005. While our evaluation primarily focuses on the implementation of the MVP’s CHW programme, we acknowledge that implementation of research knowledge alone regarding programmes such as the MVP is not sufficient to ensure that the findings will get taken up by policy [17, 18]. For this to happen, research needs to be carefully mapped to the existing political and institutional context in which policies will be implemented [18]. As a result, we enrich our evaluation by including a comparative assessment of a programme implemented in partnership with the Ministry of Health (MoH) as part of Kenya’s National CHW programme. The purpose of this is to elucidate how the MVP’s model differed to Kenya’s National CHW programme, which was under implementation at the time in a number of districts countrywide. We intend to answer the research question: what are the differences in implementation strategies between an NGO sponsored CHW programme and a MoH sponsored CHW programme? As such, our comparative analysis seeks to explore how common implementation barriers were handled by the respective programmes, which were located in adjacent villages with similar socioeconomic, demographic and health attributes [19]. As a result of this analysis, we aim to make our recommendations relevant to CHW policy-makers and programme managers working within the Kenyan national system.

## Background

### Policy context: Kenya’s National CHW programme ‘Community Strategy’

**Figure 1:**
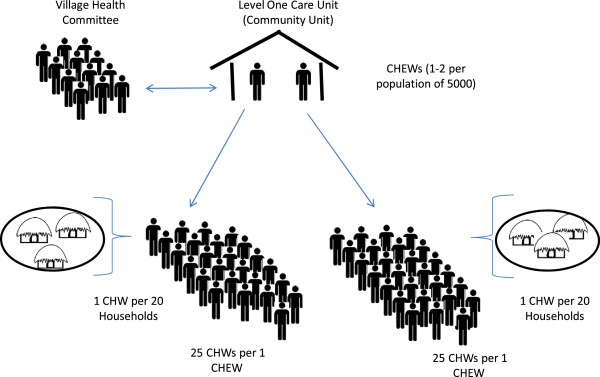
**The Kenyan community health model.** A Level One Care Unit serves a population of approximately 5,000. Between 1 and 2 trained and certified public health officers (CHEWS) each manage a cadre of 25 community health workers (CHWs), each of who are responsible for providing services to 20 households. Typically, there would be between 35 and 45 CHWs per village of 5,000. Village Health Committees work with CHEWs to mobilize and educate the community on issues of public health.

The Kenya National Health Sector Strategic Plan II (NHSSP 2005-2010) was introduced in 2005. The plan laid great emphasis on taking the Kenya Essential Package for Health (KEPH) to the community and delivering improved services to the lowest level of health service delivery through a primary health care approach [20]. Key to this strategy was the training of local community members to provide basic health services, and in so doing empowering Kenyan communities to take charge of their own health. This included the establishment of a Level One Primary Health Care Unit; a Community Unit (CU) to serve 5,000 people with a comprehensive well-trained volunteer CHW providing services to approximately 20 households. The CHWs were expected to provide basic health promotion and disease prevention services within the community. For every 25 CHWs there was to be one Community Health Extension Worker (CHEW) providing supervision and technical support. CHEWS were trained health personnel with certification in nursing or public health, and were MoH employees. Their responsibilities within the community health strategy included: facilitating trainings in the community, providing facilitative supervision to CHWs, and providing a link between CHWs and health facilities. At the same time, Community Health Committees (CHCs) were expected to organize community dialogue sessions to raise awareness of maternal and child health issues with the aid of data displayed on a community chalk board. Deliberations on community dialogue days were intended to inform the planning of community action days for health service delivery in the community. The recruitment of CHWs was to be done by each village in partnership with the CHCs (Figure 1). The strategy aimed towards reaching 16 million Kenyans or 3.2 million households [21].

Parts of the Community Strategy were revised in 2010 following resolutions of the MoH’s Health Sector Coordinating Committee (HSCC). Household coverage was revised to correspond with the population density ranging from one CHW covering 500 people for areas with dense populations in the provinces of Nairobi, Central, Western and Nyanza provinces to one CHW covering 50 people in sparsely populated areas in North Eastern province. Guidelines on contents of the CHW kit were also provided as part of the policy shift. These included basic drugs such as paracetamol, albendazole and tetracycline. The policy document also stipulated that the CHWs were entitled to a payment of KSh 2,000 (US$ 23) a month as a performance-based incentive [22].

### The Millennium Villages Project (MVP) approach

The MVP is an integrated, evidence based approach to rural poverty in sub-Saharan Africa, demonstrating the feasibility of systems delivery of health, food production, education, infrastructure and business development. The project serves as a proof of concept of accelerating progress towards the Millennium Development Goals targets. The project implements concurrent packages of MDG-focused interventions in agriculture, health, nutrition, education, water, sanitation, business development and infrastructure with an annual projected budget of US$120 per person per year sustained over a 5 to 10-year period, of which half is provided by national, regional and community partners and half is brought in by the project [23]. The interventions were recommended as important components in achieving the MDGs by the United Nations’ Millennium Project.

**Figure 2:**
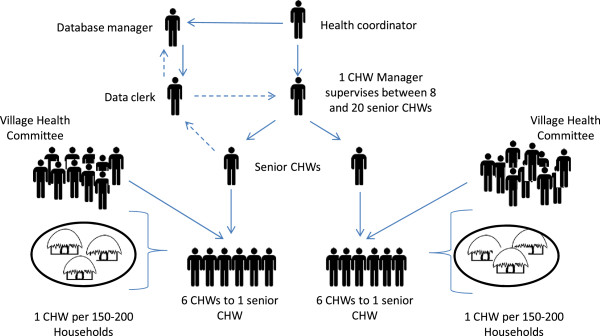
**The MVP’s CHW programme.** Total cluster size is typically between 35,000 to 70, 000, with village groupings of between 5,000 to 8,000 served by a cadre of 6 CHWs. Solid lines represent supervision, dashed lines represent flow of household health monitoring data. Monitoring data is collected by CHWs at the household level via mobile phones. Village Health Committees assist senior CHWs to monitor CHW activity at the household level.

The project commenced in 2004 in Kenya in the Western region of Kenya in a village called Bar Sauri with a population of roughly 65,000 people in what was then the Siaya District (now Gem District) [24]. The MVP’s CHW programme strategies, as well as procedures governing selection of MVs, are described elsewhere [25, 26]. In brief, the CHW programme of the MVP utilizes a workforce of CHWs, with each CHW serving at least 150 households and approximately 650 people. The MVP CHWs are supervised by senior CHWs in groups of six. The seniors are in turn supervised by Health Facilitators in a ratio of approximately 8 to 20 depending on the setting (Figure 2). The CHWs provide preventative care through health education and limited curative services. They are provided with a CHW kit that has basic drugs such as oral rehydration solution, zinc, paracetamol, Rapid Diagnostic Tests (RDTs) for malarial parasites, and Coartem for household-level treatment of positive RDT cases. The CHWs within the MVP are supported by Information and Communications Technology (ICT) systems that are facilitated through a mobile telephony system. The mobile heath technology uses information collected at the household level by CHWs to monitor child and maternal health, as well as monitor compliance with treatment administered at the clinic level. The system also prompts household visits via text message and generates feedback to CHWs and managers regarding the health status of individuals and communities.

The two CHW programmes had very different access to resources - technical, material and financial.

### Conceptual framework

**Figure 3:**
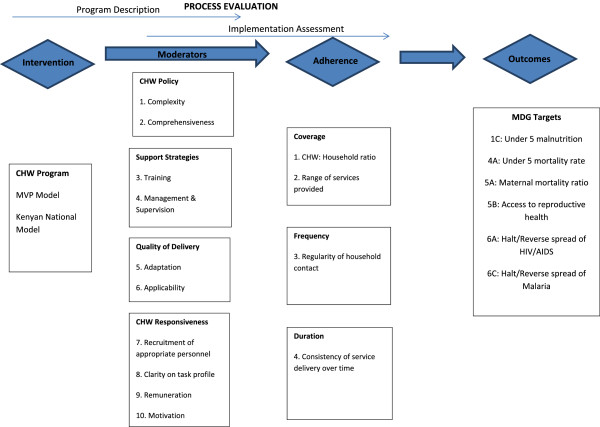
**Conceptual framework and assessment areas for the process evaluation.**

The evaluation was an implementation assessment that utilized process evaluation methods. Programme process evaluations typically attempt to verify what the programme is, as well as whether or not it is delivered as intended (or with fidelity) to the targeted recipients [27]. In the majority of studies of implementation fidelity, this is done through examining intervention adherence; in other words, the extent to which the programme delivered adheres to the programme’s design specifications with respect to coverage, frequency and duration of the intervention [28]. In addition to assessing intervention adherence, our evaluation also placed considerable emphasis on documenting the barriers and facilitators encountered when delivering the programme. These elements have been referred in the literature as intervention adherence ‘moderators’ [28], and cover a range of programme activities and attributes which include the comprehensiveness of the intervention policy; the support strategies adopted by the programme; the quality of the programme delivery; and the responsiveness of the programme participants [28]. Following the model proposed by Carroll *et al*. [28]; we used these moderators as a basis upon which to build a conceptual framework for the assessment of programme barriers and facilitators in the implantation evaluation. Within this conceptual framework, we identified ten priority assessment areas for CHW programmes which can be described as potential programme implementation moderators, and four priority assessment areas for programme adherence (Figure 3).

### Methodology

#### Study setting

The two sites were chosen purposively. The two CHW programmes were drawn from neighboring sublocations within the Gem District of Nyanza Province in the rural part of Western region of Kenya: Bar Sauri (MVP model) and Ndere (Kenyan National CHW programme model). The Bar Sauri (or ‘Sauri’) sublocation has approximately 5,000 people within Yala Division and covers 8 square km. The Ndere sublocation has a population of approximately 2,000 people and is located approximately 10 km from Sauri [24]. Household surveys conducted on 300 randomly selected households in each of the 2 central villages in 2005 also showed the villages to demonstrate similar socioeconomic, demographic and infrastructural characteristics (Table 1). Both sublocations have one health centre each. Both health centers offered outpatient services such as immunization, maternal and child health, nutrition counselling, HIV testing and antiretroviral drug (ARV) treatment. The Sauri health center also catered for normal institutional deliveries.

**Table 1:** **Key assessment areas**

		Identified assessment areas	Example evaluation questions
Implementation moderators	CHW policy	Programme complexity	How have programme designers conceptualized the CHW model? How complex and comprehensive is the model?
		Programme comprehensiveness	
	Support strategies	Training	What training did CHWs receive?
		Management and supervision	How are CHWs been monitored and supervised on the ground?
	Quality of delivery	Adaptation	In what way has the CHW model been adapted by programme managers in response to local contexts? How relevant is the current CHW model to the local context?
		Applicability	
	CHW responsiveness	Recruitment of appropriate personnel	What were the CHW recruitment processes, and how effective were they at identifying appropriate community members as CHWs?
		Clarity on task profile	
		Remuneration and Motivation	What is the understanding of CHWs of their task profiles?
			What kind of remuneration do CHWs receive?
			Are CHWs adequately motivated and incentivized to effectively perform the tasks required of them?
Implementation adherence	Coverage	CHW: Household ratio. Range of services provided	At what ratio of CHW: Household are CHWs functioning at?
			What services are actually been provided by CHWs?
	Frequency	Regularity of household contact	How frequently do CHWs visit their households?
	Duration	Consistence of service delivery over time	How consistently do CHWs deliver their required services? How consistent has been the delivery of services over time?

### Process evaluation

#### Data collection

The evaluation employed a number of methods to collect data, including a review of existing reports and records, site and facility visits, individual interviews, (n = 7), semi-structured interviews (n = 30) and focus group discussions (FGDs) (n = 6). The data was collected through a local research firm who interviewed the staff at both MVP and Ndere programmes. The evaluation questions that were used to guide interviews and focus groups are summarized in Table 1. The field work was conducted over 2 months between January and December 2011.

A total of 30 semi-structured interviews, 15 in each site, were conducted with CHWs as the key informants of the study. The sample of CHWs chosen was a convenience sample. Individual CHWs were identified as interview candidates by the Health Facilitator in the MVP site, and following a focus group with the Community Health Committee and community leaders in Ndere*.*

Each of the interviews was a minimum duration of 90 minutes and a maximum of 150 minutes. Six FGDs were also conducted, each comprising groups of at least six CHWs. Five FGDs were conducted among CHWs in the broader Sauri MVP cluster of villages. Only one FGD was conducted in Ndere due to the considerably smaller cadre of active CHWs in this site.

Additional semi-structured individual interviews were conducted with CHW supervisors known as Community Health Extension Workers (CHEWs) within the national system and their counterpart health facilitators within the MVP system. Interviews were also conducted with two CHC members in each sublocation. MoH staff within Gem District were interviewed as well as their colleagues at the national level at the Department of Community Health Services.

The evaluation design had a number of limitations and sources of potential bias, which should be stated. Firstly, the process evaluation was commissioned by the MVP and the first author served as the CHW programme manager for the MVP’s East Africa operations at the time of the evaluation. To avoid bias, she, however, did not participate in directly collecting the data. Secondly, because of the procedures for CHW selection, the Health Facilitators within the MVP might have selected CHWS who were more educated and hence more likely to provide good reports. This could attribute to some of the differences observed in the CHW profiles.

The data was collected through a local research firm who interviewed the staff at both MVP and Ndere programmes. Moreover, additional attempts were made to minimize bias by stressing anonymity and having interviews conducted in the local language by a local research firm proficient in qualitative research methodologies. Coding was also carried out independently by the first, second and third authors in an attempt to minimize sources of potential bias. Discrepancies in coding were carefully flagged and reviewed by the first author.

### Data management and analysis

The CHWs interviewed from both programmes were similar in terms of age and marital status (Table 2), with 73% and 87% of Sauri and Ndere CHWs respectively being female. The Sauri CHWs were more likely to have received secondary education.

**Table 2:** **Demographic profiles of Ndere and Sauri community health workers (CHWs) interviewed**

	Sauri CHWs (N =15) (%)	Ndere CHWs (N =15) (%)
Characteristics		
Age	34.7	38.0
Gender		
Male	4 (3)	2 (13)
Female	11 (73)	13 (87)
Marital status		
Single	2(13)	2 (13)
Married	13 (87)	13 (87)
Education level		
Primary	5 (33)	10 (67)
Secondary	8 (53)	5 (33)
Post-secondary	2 (13)	0 (0)

Interviews and FGDs were recorded by a digital voice recorder, and were later translated and transcribed into English by research assistants. A system of coding and memoing, facilitated by qualitative data analysis software (NVivo version 9, QSR International 55 Cambridge street Burlington MA 01830 USA) allowed for thematic content analysis. Pertinent excerpts were coded utilizing a coding tree that was structured in accordance with the assessment areas identified in our conceptual framework. Coding categories were defined for the data. The categories identified four main theme priority assessment areas for CHW programmes which were described as potential programme implementation moderators; CHW policy, CHW support strategies, quality of delivery of services and CHW responsiveness. Three programme adherence areas were also coded for namely: coverage, frequency and duration. Co-authors SC and MW also participated in coding independently to ensure the coding categories were consistent

### Ethical approval

The research was registered and overseen by Columbia University’s Institutional Review Board (IRB) and conformed to their guidelines for Human Subjects Research. The local IRB was also provided by the Kenya Medical Research Institute (KEMRI). All informants provided informed consent to participate in the study. Names and identifiers have been removed from all interview transcripts.

### Findings

The findings of this process evaluation are presented as follows. First, we assess the strategies adopted by each programme to encourage CHW responsiveness within each CHW policy, and critically discuss the degree to which the CHW recruitment, task profiles and models utilized by the respective programmes have responded to local challenges (quality of delivery). Following this, an analysis of implementation adherence is presented thematically in terms of the coverage, frequency and duration of delivery of services. This is followed by an analysis of implementation moderators that focus on programme support strategies. Specifically, we explore the types of support strategies offered by the respective programmes as outlined by the programme’s CHW policy, and assess the extent to which these have been successfully adapted by the programme managers to their respective local contexts.

### CHW responsiveness

#### CHW recruitment

The CHWs were generally similar in terms of age and marital status, and this was particularly reflected in the recruitment strategies utilized during selection. MVP’s CHWs responded to a job advertisement with written selection criteria, which included: ability to speak both English and Swahili; basic literacy; having come from the local village and having lived within it for the past 2 years. The announcements were made at the chief’s *baraza* (a meeting organized by the area chief to disseminate information to community members)*.* Posters with the recruitment criteria were posted within the village.

In contrast, the CHWs from the government programme in Ndere mentioned that they were recruited through a composite of the following criteria: hygiene and one’s capacity to look after the community; dedication to work without payment; empathy and concern for the sick; and general self-discipline. In addition, one’s status as a previous volunteer was also considered:

‘When we were being recruited, they didn’t look for level of education because someone can be educated but not willing to do CHWs’ work. They are only considering one’s capacity to look at the hygiene for himself and that of the community.’ (Ndere CHW Interview number 10)

The recruitment process in Ndere mainly involved Faith-Based Organizations. They sometimes used existing CHCs for mobilization and this was followed by announcements made mainly in church and at social gatherings. In one community, only those with training certificates were considered.

In both programmes, CHWs were chosen with assistance from the existing CHCs. In Sauri, Health Facilitators and some clinical staff were also involved Successful applicants from the MVP were subject to initial screening by the area and/or assistant chiefs to ensure that they were well accepted by the community. The CHCs also interviewed the applicants. They were then subjected to written as well as oral tests as a proof of their knowledge and abilities:

‘Community Health Committees (CHCs) also screened them - in order to ensure they are respected and relate well in the community. They had to be acceptable in the community*.*’ (Health Facilitator, MVP Sauri)

Recent studies show that the selection criteria for entry to a lay health worker programme determine the profile of the workers that is eventually employed [29]. Overall, the evaluation highlighted the value in a more detailed recruitment process including clear recruitment criteria and an interview process. Because MVP’s recruitment criteria for the selection of CHWs included criteria such as literacy, the MVP’s CHWs functioned well in tasks that required record keeping. The Ndere profile of CHWs, on the other hand, was based more on criteria such as ability to look after the sick and community respect. This resulted into a profile of CHWs who could mostly carry out health promotion and education. While this aligns closely to some of the policy expectation for CHWs, policy requirements such as information collection are also essential components of CHW work.

### Task profile

A common problem with CHW programmes is a lack of clarity on CHW roles [30]. This was reflected in our evaluation, with the Ndere CHWs in particular having descriptions of roles that went beyond what the MoH CHW policy had prescribed. Overall, the Ndere CHWs focused on describing health promotion activities and influencing community behavior with respect to hygiene and sanitation and bed net use, and household visitation focused on immunization compliance and referral of sick patients and children at the health facility. Ndere CHWs also described conducting general sanitation and hygiene education on the construction of dish racks, latrines, water treatment and washing hands. This was in line with the MoH formal task profile that was expected of them which called for basic preventative activities. However a few of them said they encouraged women to utilize family planning: something that was not mentioned within the official MoH CHW policy guidelines. Most of the Ndere CHWs also mentioned HIV/AIDS counselling and home-based care for people living with HIV/AIDS. These were duties that are not clarified within the MoH CHW policy.

Sauri CHWs had similar task profiles. However, more emphasis was placed on promoting door-to-door Voluntary Counselling and Testing (VCT) by trained counsellors. Sauri CHWs also described more tuberculosis (TB) management activities than Ndere CHWs. These included conducting case-detection of patients suspected to have TB and referring them for care. They also followed up on patients on Directly Observed Treatment (DOT) regimens to ensure that the TB patients who are given medication adhere to their regimens. Finally, notable differences were also reported in the tasks that CHWs from Sauri delivered to the under-five and female cohorts. The Sauri CHWs reported following up on pregnant women to ensure they received antenatal visits and made provisions for an institutional delivery. They also provided community case management for malaria using RDTs, Coartem for treatment and supporting treatment by means of MVP’s mobile telephony platform called ‘Child Count’. This platform used text messages to record treatment initiation and to advise on correct treatment follow up:

‘If two lines are indicated (on the RDT) the child has malaria. After that you send a text message to the Child Count and after the message it replies you on how you can treat the child and most likely if it is one line you do not treat you have to refer the child to the hospital because you do not know what disease the child has.’ (Sauri CHW Interview number 1)

### Remuneration and motivation

The WHO recommends that CHWs receive payment for their work or appropriate incentives [31, 32]. However, Ndere CHWs were volunteers, whereas Sauri CHWs were paid a monthly allowance of KSh 4,000 (US$ 45) by the MVP. Almost all of the Ndere CHWs felt the government should compensate them for their work. The Ndere CHWs also expressed the need to be given support and materials to do their work. The Ministry officials interviewed concurred with the Ndere CHW views:

‘If I were a supervisor, (CHWs) would be given some tokens like money, or writing materials for writing reports.’

‘The help we would prefer is remuneration. If the government can be supportive in this area of remuneration then we shall be on track.’

(Members of the Siaya District Health Management Team)

Notably, although the Sauri CHWs received a relatively generous remuneration package compared to the Ndere CHWs, many Sauri CHWs felt that remuneration was insufficient for their task profile which some asserted could not be completed on a part-time basis:

‘The tasks we do are so many and with a package of KSh 4,000, it becomes a challenge.’ (Sauri CHW Interview 6)

A district-level MoH official also indicated that the amount that was recently suggested and agreed by the government as a token; KSh 2,000 (US$ 23) - was insufficient:

*‘*I think the government has started by giving a small token, and what MVP is paying is far much ahead of what the government recommended, but we have no problem with that. Because what the Kenya government is paying and with the rate of inflation is too small. (Member of the Siaya District Health Management Team)

Interviews with national policy-makers, however, revealed the difficulties in implementing the recommended policy of providing even KSh 2,000 (US$ 22.50) to CHWs:

‘There was a policy that every constituency was to budget KSh 2,000 to allow for the recruitment of 10 CHWs. However the politicians realized that there was no mechanism in place for paying CHWs and no mechanism for distributing the money because the money was only for 10 out of 50 CHWs within the CUs. So they instead decided to use this money to pay and deploy a CHEW salary (of KSh 16,000).’ (National policy-maker from the MoH)

‘Given difficulties in rolling out payment within the government system, the Ndere CHWs claimed that the fact that they are volunteers interfered with the quality of their work, and lowered their morale. They often cited difficulties in balancing their commitment to their CHW work with livelihood activities such as farming’

‘Not being paid affects my CHW work because sometimes I should also look at how I can get money by participating in casual work so it becomes difficult to combine these two activities.’ (Ndere CHW Interview number 5)

‘Sometimes I feel that instead of taking two hours doing voluntary work, I should attend to my business. So instead of visiting the sick, sometimes I just attend to my business because we are not being paid anything.’ (Ndere CHW Interview number 7)

However, some CHWs mentioned that they sometimes are motivated by the social recognition that they received while doing their work, for instance been recognized at village gatherings such as the chief’s *baraza*:

‘ …We are also recognized during chief’s *baraza* as we are always given time to talk or just introduce ourselves… ’ (Ndere Interview number 10)

Despite these exceptions, overall the evaluation suggested that both Sauri and Ndere CHWs were primarily motivated by financial incentives. Remuneration, however, needs to be commensurate with the workload that CHWs are able to perform. The Sauri CHWs received almost double the rate suggested by the 2010 policy guidelines, but in certain contexts indicated that the remuneration that they were receiving for their work was incommensurate with the work that they were doing - particularly during scheduled campaign events aiming at universal household coverage.

### Adherence

#### Coverage, frequency and duration of service delivery

At the time of the evaluation, Ndere CHWs were still functioning on 1 CHW for every 20 households (1:20); fewer households than stipulated by the more recent policy change which required 1 CHW to cover approximately 100 households (1:100) for Nyanza province. Most Ndere CHWs said that they worked an average of 2 to 3 hours a day, usually in the afternoon. A few Ndere CHWs noted that they did not work every day and interviews with clinic supervisory staff confirmed this. One Ndere CHW noted that he worked 6 hours in a day: 3 hours in the morning and 3 hours in the afternoon and was more active during times of the year when there were fewer agricultural responsibilities:

‘I have my timetable and I do visit them (the households) when I have completed most of the day’s work. I don’t work on a daily basis. From getting up I pray, sweep, make breakfast, attend to my farm, and prepare lunch. From 2.30 pm I set off to my CHW work.’ (Ndere CHW Interview number 1)

The Sauri CHWs were functioning at a rate of 1 CHW for every 150 households a rate above the new policy stipulation that called for 1 CHW for every 500 people (approximately 100 households (1:150 > 1:100). The supervisor to CHW ratio was 1 CHW supervisor for every 35 CHWs (1:35). According to MVP policy-makers, MVP CHWs are ideally expected to be employed full-time with an average monthly salary of US$ 80 [26]. But, at the time of the evaluation, Sauri CHWs were considered part-time paid employees on a salary of US$ 45. They were expected to work 4 hours a day, 5 days a week. Most CHWs divided this workload into 2 sessions, one of around 3 hours in the afternoon and another 1 hour of work in the late evening when they wrote up their daily supervisory reports. A number of CHWs claimed working more than these hours, sometimes working full days to meet the requirements of their workload:

‘I usually take 3 hours in a day to cover all the households that I am supposed to check on, which are usually 3 homesteads. We only work for 3 hours a day. I also have a bicycle which helps me in moving around since walking on foot is usually difficult if you want to cover many homesteads.’ (Sauri CHW Interview number 8)

Amongst Ndere CHWs, tensions created by the voluntary nature of their work were the main reason cited for not carrying out tasks as intended. These tensions resulted in CHWs spending insufficient time on awareness during the chief’s *barazas* and attending to chronically ill patients. The Ndere CHWs and the district-level officials also emphasized transportation costs as hindering their coverage of household visits, as well as unrealistic expectations from community members on the ability of CHWs to assist them with some of the health challenges they face, such as transportation:

‘Sometimes I visit a patient who feels pain in the body and if I advise him/her to take an analgesic, this patient will ask me to provide for that drug thinking that I have it.’ (Ndere CHW Interview number 6)

‘The challenge I face is that some patients don’t have care givers. This sometimes forces us to visit them more often as they can’t do house work. When we visit clients they want us to support them.’ (Ndere CHW Interview number 15)

In contrast, Sauri CHWs cited challenges related to their workload and level of payment. Most CHWs claimed that their workload could only be completed if they worked full-time:

‘Some CHWs cover up to 200 households! During the month of deworming you have to go to them all - and you can do nothing else on those days - it is like you have to work full-time from 8 am in the morning to late in the afternoon. And we are not supposed to be working full-time. So really if you wanted to visit all of those households every month you would have to be working full-time.’ (Sauri CHW focus group number 3).

At the time of the evaluation, MVP was in the process of negotiating a raise in CHW salaries to ensure that remuneration was in accordance with a full-time employment package - which is indeed now the universal MVP policy [26]. It is important to note, however, that full-time employment was clearly not an option for many of the MVP CHWs. Analysis of the interview transcripts showed that all of the Sauri CHWs interviewed were also engaged in extensive household and agricultural activities. Most of the CHWs reported rising early in the morning to complete their parenting and household chores, and many of them had income generating activities such as petty trading, lumber farming, cash cropping or poultry and dairy farming. As this informant explains:

‘I can’t possibly do this work on a full-time basis. I also have other duties.’ (Sauri CHW interview number 2)

The majority of CHWs were also married women of active childbearing age (Table 2), and had family and childcare responsibilities that made the option of full-time work unattractive. Typically, however, there was a range of different opinions on this subject, and it was clear from this dialogue that at least some CHWs saw the option of full-time employment as attractive:

CHW 1: ‘We don’t care about the part-time nature of the work. If you can give me enough money I can get a maid to look after my children, and someone to work on my farm and plow my fields. I will be comfortable.’

CHW 2: ‘No, I disagree with my colleague. I do not want to hire anyone to have to do those things.’ (Sauri CHW focus group number 1)

From these interviews, it is clear that adherence to a full-time work schedule is likely to prove challenging for some CHWs. Although the MVP has now implemented a full-time work and wage policy, a priority for future evaluations would be to assess whether CHWs are adhering to this policy.

### Support strategies

#### Training

Ndere CHWs indicated that their duration of training depended on the sponsoring organization. CHWs were trained for an average of 4 weeks with a range of a minimum of 3 days and a maximum of 3 months. The training was organized at irregular intervals and the content would largely depend on the organizations responsible for funding the training. Some CHWs were trained on family planning by the German Development Agency (GTZ) and the MoH, while other CHWs were trained in water treatment by CARE. Some were trained by Comprehensive Course on Franciscan Missionary Charism (CCFMC) and St. Francis Community Development Programme (FRACODEP) on home-based care and hygiene and sanitation. Others were trained on Voluntary Counselling and Testing (VCT) and referral for ARV treatment initiation by the Centers for Disease Control (CDC).

Most Ndere CHWs expressed interest in receiving additional training to the training that they had received upon recruitment. Since not all CHWs were trained on the same topics and with the same degree of rigor, some commented on the incomplete nature of their training:

‘I just need more training on bed net use since I was only trained once.’

(Ndere CHW Interview number 4)

‘I would like more training on family planning. We only get trainings on the family planning methods that take short periods.’ (Ndere CHW Interview number 2)

Ndere CHWs stated that their training had increased their knowledge base and skills on health matters in the community, which they in turn have passed on to other community members:

‘The training has helped me gain knowledge which I use to train the community.’ (Ndere CHW Interview number 2)

However, as a policy-maker explained, implementing training programmes for CHWs within the government system has many challenges:

‘There are no regular refresher courses; the government depends on partners’ support - the partners have a certain period of time within which they operate and the partners train on specific components for CHWs to do. But this training is *ad hoc* and there is no structured programme.’

(District policy-maker at the MoH)

Sauri CHWs, on the other hand, indicated that they were trained in standardized methods for a total of 6 weeks. Four weeks were spent in classroom-style learning while the remaining 2 weeks were spent on rotation at the local Sauri Health Centre where they observed how health center staff worked. The training was fully sponsored by the MVP. Some of the training modules were conducted by the government staff:

‘We use the government curriculum. We want government to feel involved. Case management, bed nets, and water treatment - we do ourselves.’

(Sauri MVP Health Facilitator)

In contrast to the Ndere interviews, the Sauri CHWs emphasized how the training that they received allowed them to carry out referrals efficiently:

‘I have come to understand how to fill the forms. I diagnose a sick child then take them to our dispensary where we will be told if the child needs further medical attention or not. If they don’t, they sign the referral forms and take the children to Yala Level Four Hospital. This has helped me save on time.’

(Sauri CHW interview number 4)

However, some of the Sauri CHWs still expressed a desire for more training on specific topics such as counselling, family planning methods, HIV/AIDS, hygiene and sanitation, vaccination, and strategies for the protection of springs:

‘We need refresher training on how to counsel so we can know how to approach the people.’ (Sauri CHW Interview number 1)

The evaluation of both the programmes highlighted the value of a coordinated and standardized training programme for CHWs. The MVP model of utilizing training for a period of 4 to 6 weeks with components held in field, classroom and facility is in keeping with recommended guidelines for CHW programme delivery [33]. Although the Ndere CHWs had also received training from nongovernmental organizations (NGOs), interviews with government officials and CHWs both indicated that there was poor coordination between agencies and inadequate commitment to deliver training in sufficient quantities. In both programmes, CHWs indicated that they required additional training in soft skills such as counselling and more knowledge on infectious diseases. At the time of the evaluation, the MVP was in the process of revising their CHW training policy in order to meet with these emerging needs. Moving forward, the CHW National Manual may need to take into account these additional subject areas.

### Management and supervision

Ndere CHWs were supervised by a combination of the health workers based at the nearby Ndere Health Facility and members of the respective CHCs. Health facility staff met with them on a monthly basis to discuss their reports, and the supervisor oversaw their activities.

There seemed to be a good relationship between the CHWs and their facility-based supervisors:

‘We are respected at the facility by health care providers and any time we go there, they acknowledge us.’ (Ndere CHW Interview number 11)

Despite this, some CHWs mentioned that sometimes the facility-based supervisors do not spend adequate time with them working on community issues and are largely absent from the field. Such feedback is not uncommon; where lack of health personnel coupled with poorly defined supervisory tasks have been identified as factors inhibiting the delivery of effective CHW supervision [34].

The Ndere CHWs were also assisted in their tasks at the community level by the CHC members. This CHW indicates how these authorities support them in dealing with challenging issues at the community level, and raised the prestige of the CHW at community events:

‘They respect us and always share with us some challenges within the village that needs to be addressed, like a homestead without pit latrines.’ (Ndere CHW Interview 3)

When interviewed, the District Medical Officer informed us that in late 2010 the MoH re-introduced the deployment of a CHW supervisor cadre called Community Health Extension Workers (CHEWs) and each district received 10 CHEWs. However, at the time of this research, most of them had not been deployed to make household visits because they lack support such as fuel for their motorcycles to carry out adequate supervision. This national policy-maker informs us of some additional problems with using CHEWs as supervisors:

‘CHEWS do not know how to facilitate the community health workers. They work in a very top-down fashion in their management and they do not know how to engage CHWs and facilitate them to do their work better. Instead, they just give the answers. Facilitation skills are lacking. They are also not trained.’ (National policy-maker at the Ministry of Health)

The Sauri CHWs, on the other hand, were subject to a more rigorous supervision strategy. CHWs reported that they are supervised by Health Facilitators as well as the health facility staff when they work at the health facility once a week. The CHWs reported biweekly supervisory meetings as well as support in the field when needed. In the field, they mentioned that they are also overseen by the CHCs who, unlike the CHCs in Ndere, were expected to play a more active supervisory role. In focus groups and interviews, there was some evidence of tensions between the CHCs and CHWs with respects to supervision. Specifically, it was not always clear how the CHC role should be worked into supervision of CHWs, particularly given the unstructured nature of CHW work. As this dialogue between CHWs and a CHC chairman in a focus group suggests:

CHW #1: ‘The community health committees do not understand the work of the CHWs. They do not understand our rosters or the fact that we work on flexible timing. They accuse you of not doing your work. They don’t understand that you might have worked long hours a day before in order to have some free time for some other activities’.

CHC chairman: ‘We have said it right from the beginning. You cannot work in isolation. Sometimes it is obvious that the person is supposed to be doing their work. If you know you are going to be away they should have to inform the committees that you are going away!’

CHW #2: ‘There could be someone sick in the home. What will happen then? Are we supposed to ask permission from the committees for our every move?’

CHW #1: ‘We need clear guidelines. How many days are we supposed to be in the community? What is the expectation? So we can be monitored in the system they understand.’ (Sauri CHW focus group number 1)

In contrast, with respects to supervision by MVP Health Facilitators, none of the Sauri CHWs interviewed reported negatively on the quality of supervision they received, although informants may have been reluctant to express criticism due to the internal nature of the evaluation:

‘Our relationship is good because we listen to what they say and do what they ask us to do. I have never been in bad terms with them. We receive the expected level of supervision because if we underperform in an area, they sit us down and explain to us what is expected of us. We then redo the work as expected.’ (Sauri CHW Interview number 8)

The MVP Health Facilitators utilized mobile telephony as a supervisory tool. The Child Count rapid SMS system provides alerts to Health Facilitators when CHWs have difficult cases:

‘Every day I get alerts. I look at the information, and if it is case management (that is malaria) if it is negative I follow up with the CHW and make sure that they have sent to the clinic for diagnosis. Then diarrhoea I ensure follow up after one day and if it is not better - they are referred to clinics. And the nutrition clinic, if they are not attending I make sure there is a home visit scheduled. I actually phone the CHW every day. I get between 10 to 30 alerts a day.’ (Health Facilitator, MVP Sauri)

The CHWs describe how their supervisors carry out supervision through the use of the toll-free numbers, which their supervisors use to call them and discuss challenges while they are making their household visits and hence removing the need to be physically present with the CHWs:

‘They normally call us and arrange meetings with us and advise us on what to do. We have these toll-free phones so that if we have any issue, we just call them and explain the situation to them. They then advise us on what to do.’ (Health Facilitator, Sauri)

## Conclusion

CHWs have the potential to increase access to and coverage of basic health services. However, programme managers and policy-makers need to pay close attention to the details in implementation of CHW programmes if intended outcomes are to be achieved. Through comparing two different CHW programmes in neighboring villages, we highlight how the range of services offered by CHWs can vary with the implementation strategy. Prior evaluations of CHW programmes have typically recommended that for CHW programmes to be effective appropriate recruitment, adequate remuneration, strong supervision and support are essential [5, 9, 35]. Our evaluation revealed a number of ways in which effectiveness of CHW programmes within these areas could be improved.

Firstly, programme managers and MoH programmes need to pay closer attention to the recruitment processes that they utilize. The criteria for selection of CHWs need to be clearly stated in writing and the candidate profiles need to be predetermined prior to selection. While there is merit in favouring candidates with specific community traits, such as volunteerism, the evaluation suggested that certain minimum criteria need to be adhered to. Moreover, human resource processes, such as interviews, are useful for assessing soft and hard skills before CHW selection is confirmed. It was particularly useful when these more formal processes followed initial screening at, for example, a chief’s *baraza*.

Secondly, the evaluation suggested that the national policy might benefit from a clearer job description and scheme of services for CHWs. It would be additionally helpful if the typology of the CHWs was stated in writing before commencement of programmes. This would spell out whether the CHWs to be recruited are generalist CHWs who provide basic health promotion and disease prevention or whether they are CHWs who will be trained further in case management and provide some curative services. CHWs should be given guidelines and clear terms of reference that enumerate clearly both preventative and curative tasks that they should undertake within the community. For curative tasks, the tools of work and the drugs that they provide should be made available as per the CHW kit defined by the CHW policy.

Thirdly, CHWs within both NGO and national programmes need to be remunerated a living wage to ensure motivation to the tasks required and to avoid attrition from programmes. However, it was clear from this evaluation that there appear to be significant challenges in implementing the 2010 policy of the Kenyan Government in offering CHWs a KSh 2,000 (US$ 22.50) stipend in the field. Options for community payment had not been considered because of the poverty levels within this district, and an earlier study by Ofusu-Ammah identifies community payment as being irregular and can lead to higher attrition [35]. Clearly, financing modalities for this policy position need to be urgently catered for by the MoH through their donors to make implementation of Community Strategy a reality. Although a number of factors can lead to attrition, including poor selection criteria, lack of adequate support and the cultural environment, remuneration is a powerful factor in ensuring retention of CHWs. Countries such as Sri Lanka have previously utilized volunteer CHW programmes but have experienced high attrition rates making the programmes unsustainable [36]. Moreover, whereas hope of eventual remuneration may well be a strong motivation for volunteers to join CHW programmes [37], it does not seem to stop CHWs from leaving [38]. When present, attrition was usually attributable to a lack of prospects for CHWs to grow professionally within the system and the inability to balance the workload and personal commitments. Better integration of a model that proposes a living wage or offering prospects for professional growth within the health sector should be explored [39].

Fourth, the important question of the optimal population size that a CHW can cover needs to be rethought [40]. While coverage of households may be predetermined by policy, the reality is that in practice population size, typology and the availability of means of transport should guide the number of households covered by CHWs during their visits. From interviews with Sauri CHWs, at the time of the evaluation the target ratio assigned by the MVP of 1 CHW: 150 households was only feasible for a CHW working on a part-time basis when campaign events (such as door-to-door growth monitoring and deworming) were not underway. This was an important limitation of the MVP model, and a source of considerable conflict between CHWs and their supervisors. The lessons learned from this process suggest that ideally, CHW task loads should be established during recruitment with CHWs incentivized for extra duties performed outside of their regular task loads.

Fifthly, with respect to supervision, most studies have identified lack of supervision and support has one of the biggest problems in CHW programme implementation [5, 6, 9]. On the whole, innovations such as the use of mobile telephony solutions to issue alerts to supervisors appeared to strengthen CHW supervision, as did the MVP’s policy towards frequent (biweekly) supervisory meetings and regular contact with CHWs in the field. Moreover, the existence of toll-free communication lines between CHWs and CHW supervisors in the MVP site was highly effective in monitoring CHW activities. Clearly, although many inroads have been made towards strengthening supervision within national programmes, there are a number of problems remaining. In addition to this, essential elements such as deployment of CHEWs, transport costs of CHW supervisors, areas of coverage and supervisory tasks of the CHW supervisors would benefit from clearer definition by the national policy.

Sixth, our evaluation further emphasized the value of training, which is performed in a regular fashion ideally at a period of 4 to 6 weeks with components held in field, classroom and facility [33]. For CHWs in the government programmes, this approach may benefit from being standardized using a MoH National Manual. The evaluation further suggested that NGOs implementing the national programme might benefit from more coordinated CHW trainings with the District Teams so that they prioritize pre-service training for CHWs as opposed to specialized areas of their own organizations interest.

Moreover, in both programmes the role of the CHC needs to be carefully considered. While CHCs seemed to provide a useful link during recruitment and community facilitation, their role in supervision of CHWs is less clearly defined. The MVP, in particular, tried to implement a policy of active CHW supervision by CHCs at the household level, but in practice the feedback process between CHWs, CHW supervisors and CHCs was difficult to harmonize. This limitation has been recognized by the MVP, who has put considerable efforts into better refining the CHC supervision policy. Clearly, more needs to be done to utilize the CHCs as a governance tool in CHW programming.

In summary, both the MVP and national CHW programmes both faced considerable challenges in implementation. Due to better flexibility, resources and scope for rapid innovation on the ground, the MVP model was able to introduce a number of innovations that aimed to strengthen CHW management, supervision and improve CHW responsiveness. Many of these innovations proved very effective in smoothing programme operations, but programme adherence still faced a number of challenges with respects to ensuring that CHW coverage was adequate, visitation frequency was sufficient and services were delivered with the same consistency over time by all CHWs. Since conducting the evaluation, MVP programme staff has continued to adapt the MVP policy in an effort to address these challenges, and all MVP CHWs are now paid a salary that is commensurate with a full-time wage [26].

Adherence to this model, however, may still prove a challenge for some CHWs as CHWs typically hold numerous other responsibilities in the community, which they may be unwilling or unable to relinquish. This is an ongoing challenge for many CHW programmes, and is best addressed on a case-by-case basis by the policy-makers and programme managers both from the government and NGOs implementing these programmes on the ground. Further implementation research in this area will be a priority area for future evaluations, as CHW cadres become increasingly professionalized and these activities are scaled up throughout sub-Saharan Africa [41].

## Abbreviations

ARV: Antiretroviral Therapy
CCFMC: Comprehensive Course on Franciscan Missionary Charism (CCFMC)
CDC: Centre for Disease Control
CHC: Community Health Committee
CHEW: Community Health Extension Worker
CHW: Community Health Worker
CU: Community Unit
DOT: Directly Observed Treatment
FGD: Focus Group Discussion
FRACODEP: St. Francis Community Development Program
GTZ: German Development Agency
HIV/AIDS: Human Immunodeficiency Virus/ Acquired Immune Deficiency Syndrome
IRB: Institutional Review Board
MOH: Ministry of Health
MVP: Millennium Villages Project
NHSSP: National Health Sector Strategic Plan
NGO: Non-Governmental Organization
SMS: Short Messaging Service
RDT: Rapid Diagnostic Testing
TB: Tuberculosis
VCT: Voluntary Counselling and Testing
WHO: World Health Organization.

## References

[1] Lehmann U, Sanders D (2007). Community Health Workers: What do we Know About Them? The State of the Evidence on Programs, Activities, Costs and Impacts on Health Outcomes of Using Community Health Workers.

[2] Declaration of Alma-Ata (1978). Adopted at International Conference on Primary Health Care; 6 to 12 September.

[3] Lewin S, Dick J, Pond P, Zwarenstein M, Aja G, van Wyk B, Bosch-Capblanch X, Patrick M (2005). Lay health workers in primary and community health care. Cochrane Database Syst Rev.

[4] Walt G (1990). Community Health Workers in National Programmes. Just Another Pair of Hands?.

[5] Berman PA, Gwatkin DR, Burger SE (1987). Community based health workers: head start or false start towards health for all*?*. Soc Sci Med.

[6] Haines A, Sanders D, Lehmann U, Rowe AK, Lawn JE, Jan S, Walker DG, Bhutta Z (2007). Achieving child survival goals: potential contribution of community health workers. Lancet.

[7] Stekelenburg J, Kyanamina SS, Wolffers I (2003). Poor performance of community health workers in Kalabo District, Zambia. Health Pol J.

[8] Finau SA, Tamoepeau B, To’a L (1986). Review of the village health worker pilot scheme in Tonga. N Z Med J.

[9] Gilson L, Walt G, Heggenhougen K, Owuor-Omondi L, Perera M, Ross D, Salazar L (1989). National community health worker programs: how can they be strengthened?. J Pub Health Pol.

[10] Walt G, Ross D, Gilson L, Owuor-Omondi L, Knudsen T (1989). Community health workers in national programmes: the case of the family welfare educators of Botswana. Trans Royal Soc Trop Med Hyg.

[11] Initiative TJL (2004). Human Resources for Health: Overcoming the Crisis.

[12] Van Lerbergh W (2008). The World Health Report 2008: Primary Health Care: Now More Than Ever.

[13] Bhutta ZA, Lassi ZS, Pariyo G, Huicho L (2010). Global Experience of Community Health Workers for Delivery of Health-Related Millennium Development Goals: A Systematic Review, Country Case Studies and Recommendations for Scaling up.

[14] Sanders D, Haines A (2006). Implementation research is needed to achieve international health goals. PLoS Med.

[15] Zachariah R, Ford N, Maher D, Bissell K, Van den Bergh R, van den Boogaard W, Reid T, Castro KG, Draguez B, von Schreeb J, Chakaya J, Atun R, Lienhardt C, Enarson DA, Harries AD (2012). Is operational research delivering the goods? The journey to success in low-income countries. Lancet Infect Dis.

[16] Daniels K, Nor B, Jackson D, Ekström EC, Doherty T (2010). Supervision of community peer counselors for infant feeding in South Africa: an exploratory qualitative study. Hum Res Health.

[17] Whitty C, Kinn S (2011). Foreword: lesson learning about getting research into policy and practice. Health Res Pol Syst.

[18] Panisset U, Koehlmoos TP, Alkhatib AH, Pantoja T, Singh P, Kengey-Kayondo J, McCutchen B, Ángel Miguel G (2012). Implementation research evidence uptake and use for policy-making. Health Res Pol Syst.

[19] Pronyk PM, Muniz M, Nemser B, Somers MA, McClellan L, Palm CA, Huynh UK, Amor YB, Begashaw B, McArthur JW, Niang A, Sachs SE, Singh P, Teklehaimanot A, Sachs JD (2012). The effect of an integrated multisector model for achieving the Millennium Development Goals and improving child survival in rural sub-Saharan Africa: a non-randomized controlled assessment. Lancet.

[20] Ministry of Health (2005). Reversing the Trends: The Second National Health Sector Strategic Plan of Kenya (NHSSP II) 2005–2010.

[21] Ministry of Health (2006). Taking the Kenya Essential Package of Health to the Community. A Strategy for the Delivery of Level one Service.

[22] Director of Public Health and Sanitation (2011). Policy Shift on Community Strategy.

[23] Sanchez P, Palm C, Sachs JD, Denning G, Flor R, Harawa R, Jama B, Kiflemariam T, Konecky B, Kozar R, Lelerai E, Malik A, Modi V, Mutuo P, Niang A, Okoth H, Place F, Sachs SE, Said A, Siriri D, Teklehaimanot A, Wang K, Wangila J, Zamba C (2007). The African millennium villages. PNAS USA.

[24] Mutuo P, Lelerai E, Okoth H, Oule J, Ouma BA, Wangila J (2006). Baseline Report Sauri, Millennium Research Village.

[25] Liu A, Sullivan S, Khan M, Sachs S, Singh P (2011). Community health workers in global health: scale and scalability. Mt Sinai J Med.

[26] McCord GC, Liu A, Singh P (2012). Deployment of community health workers across rural sub-Saharan Africa: financial considerations and operational assumptions. Bull World Health Org.

[27] Rossi P, Lipsey M, Freeman H (2004). Evaluation: A Systematic Approach.

[28] Carroll C, Patterson M, Wood S, Booth A, Rick J, Balain SA (2007). A conceptual framework for implementation fidelity. Implement Sci.

[29] Nkonki L, Cliff J, Sanders D (2011). Lay Health Worker attrition: important but often ignored. Bull World Health Org.

[30] Matomora MK (1998). Mass produced VHWs and the promise of primary health care. Soc Sci Med.

[31] World Health Organization: *Scaling up, Saving Lives*. Geneva: WHO;

[32] World Health Organization (2007). Task Shifting: Rational Redistribution of Tasks Among Health Workforce Teams.

[33] Bhutta ZA, Pariyo G, Huchi L (2010). Global Experience of Community Health Workers for Delivery of Health Related Millennium Development Goals: A Systematic Review, Country Case Studies and Recommendations for Integration into National Health Systems.

[34] Ojofeitimi EO, Jinadu MK, Elegbe I (1998). Increasing the productivity of community health workers in rural Nigeria through supervision. Soc Econ Plan Sci.

[35] Ofusu-Ammah V (1998). National experience in the use of Community Health Workers. A review of current issues and problems. WHO Offset Publ.

[36] Walt G, Perera M, Heggenhougen K (1989). Are large-scale volunteer community health worker programmes feasible? The case of Sri Lanka. Soc Sci Med.

[37] Kironde S, Klasse S (2002). What motivates lay volunteers in high burden but resource-limited tuberculosis control programmes? Perceptions from the Northern Cape Province, South Africa. Int J Tuberc Lung Dis.

[38] Olan’go CO (2010). Staff attrition among CHWs in home based care programmes for people living with HIV and AIDS in Western Kenya. Health Pol.

[39] Glenton C, Scheel IB, Prahdan S, Lewin S, Hodgins S, Shrethsa V (2010). The female community health volunteer in Nepal decision makers’ perceptions of volunteerism, payment and other incentives. Soc Sci Med.

[40] Prasad BM, Muraleedharan VR (2007). Community Health Workers: A Review of Concepts, Practice and Policy Concerns.

[41] Singh P, Sachs J (2013). 1 million community health workers in sub-Saharan Africa by 2015. Lancet.

